# Zika virus alters the microRNA expression profile and elicits an RNAi response in *Aedes aegypti* mosquitoes

**DOI:** 10.1371/journal.pntd.0005760

**Published:** 2017-07-17

**Authors:** Miguel A. Saldaña, Kayvan Etebari, Charles E. Hart, Steven G. Widen, Thomas G. Wood, Saravanan Thangamani, Sassan Asgari, Grant L. Hughes

**Affiliations:** 1 Department of Microbiology and Immunology, University of Texas Medical Branch, Galveston, TX, United States of America; 2 Australian Infectious Disease Research Centre, School of Biological Sciences, The University of Queensland, Brisbane, Australia; 3 Department of Pathology, University of Texas Medical Branch, Galveston, TX, United States of America; 4 Department of Biochemistry and Molecular Biology, University of Texas Medical Branch, Galveston, TX, United States of America; 5 Department of Pathology, Institute for Human Infections and Immunity, Center for Tropical Diseases, Center for Biodefense and Emerging Infectious Disease. University of Texas Medical Branch, Galveston, TX, United States of America; The Connecticut Agricultural Experiment Station, UNITED STATES

## Abstract

Zika virus (ZIKV), a flavivirus transmitted primarily by *Aedes aegypti*, has recently spread globally in an unprecedented fashion, yet we have a poor understanding of host-microbe interactions in this system. To gain insights into the interplay between ZIKV and the mosquito, we sequenced the small RNA profiles in ZIKV-infected and non-infected *Ae*. *aegypti* mosquitoes at 2, 7 and 14 days post-infection. ZIKA induced an RNAi response in the mosquito with virus-derived short interfering RNAs and PIWI-interacting RNAs dramatically increased in abundance post-infection. Further, we found 17 host microRNAs (miRNAs) that were modulated by ZIKV infection at all time points. Strikingly, many of these regulated miRNAs have been reported to have their expression altered by dengue and West Nile viruses, while the response was divergent from that induced by the alphavirus Chikungunya virus in mosquitoes. This suggests that conserved miRNA responses occur within mosquitoes in response to flavivirus infection. This study expands our understanding of ZIKV-vector interactions and provides potential avenues to be further investigated to target ZIKV in the mosquito host.

## Author summary

Vector-borne viruses have immense impacts on human health by causing mortality and morbidity. Control of diseases caused by these viruses have mostly concentrated on vector control or inhibition of virus transmission by the vectors. This requires a thorough understanding of vector-virus interactions. In this study, we investigated the RNA interference (RNAi) response in *Aedes aegypti* mosquitoes infected with the Zika virus (ZIKV) strain isolated from the current pandemic using deep sequencing technologies. We found that infection alters the microRNA (miRNA) profile between infected and uninfected mosquitoes and that changes in miRNA expression occur over time. The short interfering RNA pathway, which is the main mosquito defense as part of the RNAi pathway, was also induced by ZIKV infection with the number of short interfering RNAs increasing significantly as infection progressed. Our results indicate that ZIKV induces the mosquito host defense similar to infection with other flaviviruses.

## Introduction

Zika virus (ZIKV) is a flavivirus related to dengue virus (DENV), West Nile virus (WNV) and Yellow fever virus (YFV) that is transmitted to humans by *Aedes* mosquitoes. In the urban transmission cycle, *Aedes aegypti* is thought to be the dominant vector, while several *Aedes* species are implicated in transmission in the sylvatic cycle [1,2]. The virus was originally discovered in the Ziika forest in Uganda [3] and has likely been circulating in monkey and human populations in Africa and Asia. In the last 10 years, an Asian virus lineage has rapidly spread on an unprecedented timescale around the pacific and the Americas. In humans, the neurotropic virus causes microcephaly in newborns and has been implicated in other neurological disorders such as Guillain-Barre syndrome [4]. The explosive spread of the virus and its effect on infants created a public health emergency and stimulated research efforts to investigate new treatments and vaccines to reduce these conditions. Although significant progress has been achieved concerning the interaction of ZIKV with the mammalian host since the outbreak, we still have a poor understanding of the molecular interplay between the virus and the mosquito host. As vector control is the only viable option for alleviating the diseases caused by ZIKV, a more thorough understanding on these interactions is critical.

Arbovirus infection of mosquitoes elicits complex interactions between the host and the virus. In some cases, the mosquito’s innate immune pathways, which can be antagonistic to viral infection, are provoked by arboviruses. However, these immune pathways appear to be virus specific as the Toll and JAK-STAT pathways are antagonistic to DENV yet do not appear to influence other arboviruses such as Chikungunya virus (CHIKV) or ZIKV [5–8]. In addition to these classical immune pathways, RNA interference (RNAi) and microRNAs (miRNAs) are important components that dictate host-microbe interactions for arboviruses and their mosquito vectors [9–11]. PIWI-interacting RNAs (piRNAs), another group of noncoding small RNAs of 25–30 nt, could also potentially be involved in arbovirus-mosquito interactions [12].

miRNAs are small non-coding RNAs (~22 nt) that regulate gene expression post transcriptionally. In mosquitoes, miRNAs are important in many developmental processes and nutrition [13,14] and it is becoming clear that these molecules are critical in host-pathogen interactions [9,10,15]. Several studies have shown that pathogen infection alters the miRNA expression profile in mosquitoes (reviewed in [11]). This alteration could be due to the host responding to the pathogen or by the pathogen attempting to alter gene expression in the host to make its environment more suitable. For example, the mosquito-borne alphavirus North American eastern equine encephalitis virus (EEEV) alters a host miRNA to avoid the host’s immune response [16]. In *Ae*. *aegypti*, infection with DENV alters the miRNA profile [17], with temporal variation in miRNA expression observed with 23 miRNAs altered at 9 day post infection (dpi) compared to five or less at 2 and 4 dpi. In the Asian tiger mosquito, *Aedes albopictus*, the miRNA, miR-252, increased after a DENV infected blood meal, and inhibition of this miRNA resulted in increased viral copies while overexpression of the miRNA suppressed virus [18]. Taken together, these studies demonstrate that miRNAs can contribute to the complex interactions occurring between invading arboviral pathogens and their mosquito host, and that this interplay likely dictates vector competence.

While our understanding of these pathways on arbovirus vector competence is expanding, there is a dearth of knowledge related to how ZIKV may alter the miRNA profile in the vector or the human host. To address this issue, here we used high throughput sequencing to examine the small RNA profiles after viral infection of the primary ZIKV vector *Ae*. *aegypti*. We examined host miRNA, virus-derived short interfering RNA (viRNA) and piRNA profiles at various time points post-infection. Our results provide the first molecular evidence that infection of ZIKV alters the miRNA profile of a host and the mosquito host mounts an RNAi response against the virus.

## Methods

### Ethics statement

The ZIKV strain was acquired from the World Reference Center for Emerging Viruses and Arboviruses at the University of Texas Medical Branch (Galveston, TX, USA). The virus was originally isolated from an *Ae*. *aegypti* mosquito (Chiapas State, Mexico). ZIKV protocols were approved by the University of Texas Medical Branch Institutional Biosafety Committee (Reference number: 2016055).

### Mosquito infections with Zika virus

Four-six day old female *Ae*. *aegypti* (Galveston strain) mosquitoes were orally infected with ZIKV (Mex 1–7 strain) at 2 x 10^5^ focus forming units (FFU)/ml) in a sheep blood meal (Colorado Serum Company). At 2, 7 and 14 days post-infection (dpi) RNA was extracted from whole mosquitoes using the mirVana RNA extraction kit (Life Technologies) following the protocol for extraction of total RNA. Viral infection in mosquitoes was confirmed by Taqman qPCR on ABI StepOnePlus machine (Applied Biosystems) using a ZIKV-specific probe and primers (S4 Table). RNA from ZIKV positive samples was pooled (N = 5) for time points 7 and 14. Limited ZIKV positive samples were detected at day 2, likely due to the virus titer being at the limits of detection for qPCR. For this time point, at least 1 qPCR positive individual was included in each pool. For all time points, three independent pools were used to create libraries for infected and uninfected samples. Control mosquitoes were fed with blood devoid of ZIKV and collected at the same time points and processed in the same way as infected ones.

### Library preparations and sequencing

Small RNA libraries were created using the New England Biolabs small RNA library protocol (New England Biolabs). Library construction used a two-step ligation process to create templates compatible with Illumina based next generation sequence (NGS) analysis. Where appropriate, RNA samples were quantified using a Qubit fluorometric assay (Thermo Fisher Scientific). RNA quality was assessed using a pico-RNA chip on an Agilent 2100 Bioanalyzer (Agilent Technologies). Library creation uses a sequential addition of first a 3’ adapter sequence followed by a 5’ adapter sequence. A cDNA copy was then synthesized using ProtoScript reverse transcriptase (New England Biolabs) and a primer complimentary to a segment of the 3’ adapter. Amplification of the template population was performed in 15 cycles (94°C for 30 sec; 62°C for 30 sec; 70°C for 30 sec) and the amplified templates were PAGE (polyacrylamide gel electrophoresis) purified (147 bp DNA) prior to sequencing. All NGS libraries were indexed. The final concentration of all NGS libraries was determined using a Qubit fluorometric assay and the DNA fragment size of each library was assessed using a DNA 1000 high sensitivity chip and an Agilent 2100 Bioanalyzer. Sequence analysis was performed using the rapid run platform and single end 50 base sequencing by synthesis on an Illumina Hi-Seq 1500 using the TruSeq SBS kit v3.

### Small RNA analysis

CLC Genomic Workbench (version 7.5.1) was used to remove adapter sequences and reads with low quality scores from datasets. We applied the quality score of 0.05 as cut off for trimming. As described in CLC Genomic Workbench manual the program uses the modified-Mott trimming algorithm for this purpose. The Phred quality scores (Q), defined as: *Q = -10log*_*10*_*(P)*, where P is the base-calling error probability, can then be used to calculate the error probabilities, which in turn can be used to set the limit for which bases should be trimmed. Reads without 3’ adapters or with less than 16 nt were also discarded from the libraries. Clean data were considered as mappable reads for further analysis. We used small RNA tool in CLC Genomic Workbench to extract and count unique small RNA reads with minimum five sampling count. Tab separated files with the read sequences and their counts were used as input file for novel and homologous miRNA analysis using sRNAtoolbox [19]. All known *Ae*. *aegypti* precursor miRNAs reported in miRBase 21 were used as reference for miRNA annotation [20]. The ultrafast short read aligner Bowtie was used to align the reads to the *Ae*. *aegypti* genome and the miRNA database. The alignment type “n” was selected and we allowed a maximum of one mismatch in the Bowtie seed region for genome, and known and homologous miRNA database in our mapping parameters. The seed alignment length for Bowtie was 20 for all the analyses. Differential expression of miRNAs between two conditions was calculated and normalized based on the DESeq package with EdgeR [21] on sRNAtoolbox server, and final fold change values were given in log_2_ scale.

### RNAi activity analysis

To understand the RNAi activity against ZIKV, we mapped all the small RNAs to the viral genome (Accession No. KX247632). We implemented strict mapping criteria (mismatch, insertion and deletion costs: 2: 3: 3, respectively). The minimum similarity and length fraction of 0.9 between a mapped segment and the reference were allowed in mapping criteria. We ignored reads with more than one match to viral genome in mapping parameters. Mappable reads in all libraries were filtered and only reads with 21 nt in length were selected to check their mapping pattern to negative and positive strands of the virus genome. We also sorted all mappable reads between 25–30 nt to the viral genome for checking any potential piRNA signature.

### miRNA target identification

We used three different algorithms including RNA22 [22], miRanda [23] and RNAhybrid [24] to predict potential miRNA binding sites in all the *Ae*. *aegypti* annotated genes (GCF_000004015.3_AaegL2) and ZIKV genome (KX247632). The small RNA sequence was hybridized to the best fitting portion of the mRNA or viral genome by RNAhybrid. We did not allow G:U pairing in the seed region (nucleotides 2–8 from the 5’ end of the miRNA) and forced miRNA-target duplexes to have a helix in this region. Maximum 5 nt were approved as unpaired nucleotides in either side of an internal loop. miRanda also considers matching along the entire miRNA sequence but we ran the program in strict mode which demands strict 5’ seed region (nucleotide 2–8 from the 5’ end) pairing. It takes the seed region into account by adding more value to matches in the seed region. RNA22 v2 is a pattern based target prediction program which first searches for reverse complement sites of patterns within a given mRNA sequence and identifies the hot spots. In the next step, the algorithm is searched for miRNAs that are likely to bind to these sites. We allowed maximum 1 mismatch in the seed region and minimum 12 nt matches in the entire binding site. We set the sensitivity and specificity thresholds to 63% and 61%, respectively. miRNA binding sites on *Ae*. *aegypti* mRNAs, which were predicted by all the three algorithms are considered as highly confident miRNA binding sites.

### RT-qPCR analysis of miRNAs

RNA samples were converted to cDNA using a miSCRIPT II RT kit (Qiagen) using the HiSpec buffer to assure that the cDNA produced was derived only from mature miRNA molecules. 5μL of RNA was used per reaction with an average 605ng per sample. One additional reaction was prepared with no RNA template. The reaction was heated on a Mastercycler-Pro thermal cycler (Eppendorf). Real-time PCR was performed using an IQ5 cycler (BioRad) and with Quantitech SYBR master mix (Qiagen). The process was performed using the proprietary-sequence universal primer provided with the kit as the reverse primer and 10 μM of one of nine miRNA-specific forward primers (IDT), the sequence of which is listed in S4 Table. The cDNA was diluted with 60 μL of nuclease-free water per 30 μL of RT product solution, and 2 μL of diluted cDNA was used per reaction. The volumes of the master mix and primers used were those recommended by their manufacturer. Each sample was run in duplicate and the Ct values averaged for further mathematical processing. The amplification program began with 95°C for 15min, followed by forty cycles of 94°C for 15s, 55°C for 30s, and 70°C for 30s. Gene expression analysis was performed using the ΔΔCt (Livak) method [25]. The miRNA expression in each sample was normalized to the expression of U6B small nuclear RNA (RNU6B). Our RT-qPCR results confirmed that U6B remained quite stable across infected and non-infected samples (S1 Fig). For each day, six RNA samples were used: three from mock-infected mosquitoes, and three from ZIKV-infected mosquitoes. For each day post-infection, individual ΔCt values for both mock and ZIKV samples were used to calculate relative difference of expression. “No-template” controls were included on each plate run.

### Accession numbers

The accession number for the raw and trimmed sequencing data reported in this paper is GEO: GSE97523.

## Results and discussion

### Deep sequencing of small RNAs

**Fig 1:**
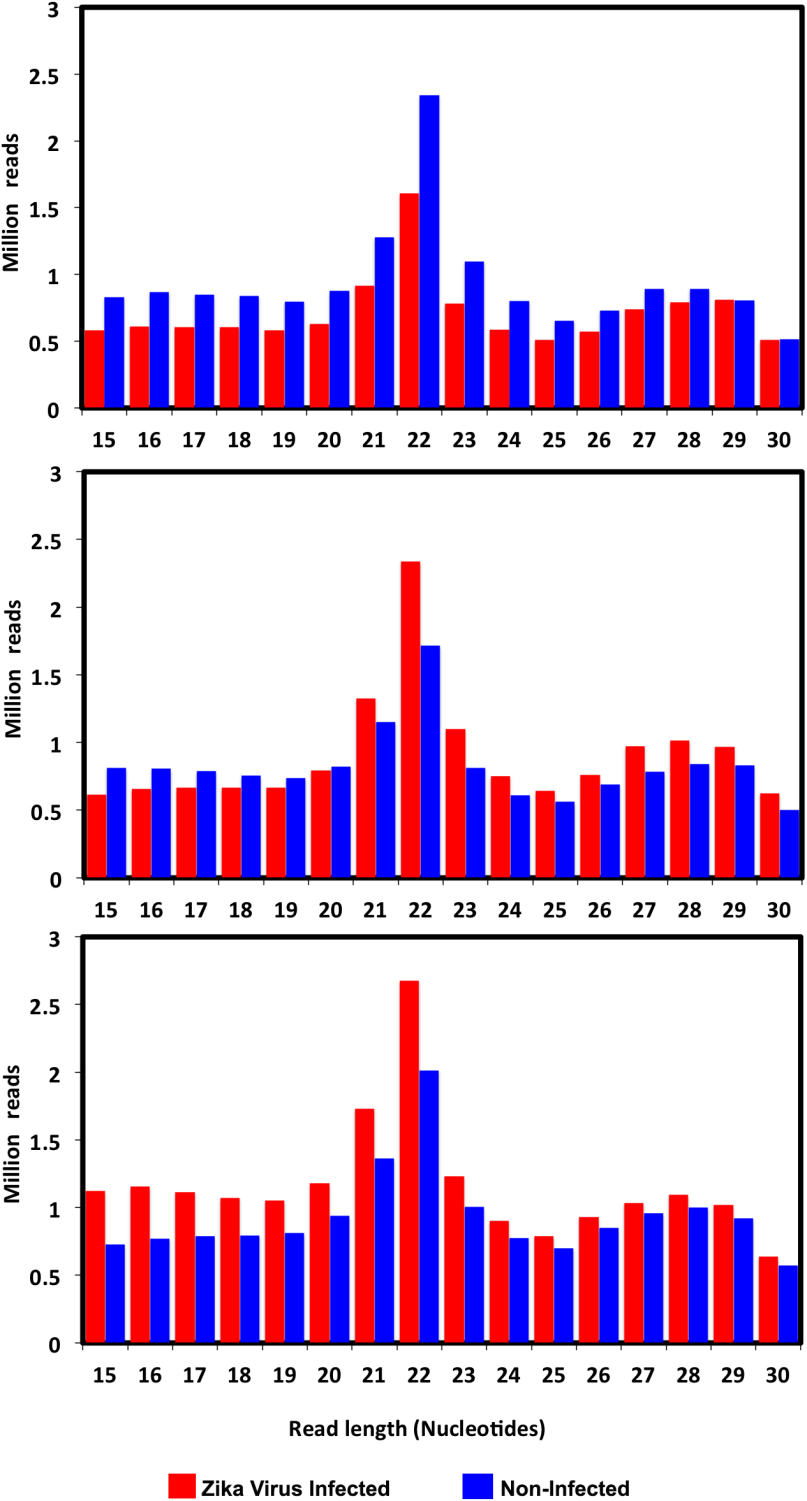
Length distribution of mappable reads to the *Ae*. *aegypti* genome obtained from ZIKV-infected and non-infected mosquitoes at day 2, 7 and 14 post-inoculation.

Illumina small RNA deep sequencing platform was used to produce small RNA profiles in ZIKV-infected and non-infected *Ae*. *aegypti* mosquitoes. RNA samples were extracted from whole mosquitoes collected at 2, 7 and 14 days post-infection (dpi) to explore host miRNA and RNAi responses to ZIKV infection. ZIKV infection was confirmed in individual mosquitoes by RT-qPCR, which indicated increases in viral load as infection progressed (S2 Fig). We obtained 59.5–61.8 million combined raw reads from the non-infected libraries in day 2, 7 and 14 samples, respectively (S1 Table). From ZIKV-infected libraries, 54.7–84.8 million reads were acquired after combining all the three biological replicates in day 2, 7 and 14 post-infection, respectively (S1 Table). 15–25% of reads were discarded in different libraries due to their low-quality score or lack of adapter sequence. We detected most of the annotated *Ae*. *aegypti* miRNAs present on miRBase in our data representing 10–17% of clean reads in different libraries. In all libraries, total read numbers over different lengths showed a peak at 21–22 nucleotides (nt) representing the typical length of miRNAs and short interfering RNAs (siRNAs) (Fig 1). Another smaller peak at 27–29 nt was obtained probably pertaining to PIWI-interacting RNAs (piRNAs), which are common in most insect small RNA libraries.

### Differential expression of *Ae*. *aegypti* miRNAs in response to ZIKV infection

Small RNA libraries from ZIKV-infected *Ae*. *aegypti* mosquitoes showed alteration of miRNA profiles compared with non-infected controls at 2, 7 and 14 dpi. However, only 17 miRNAs were identified as differentially modulated at all the time points, with the majority of them significantly depleted in response to ZIKV infection (Table 1). At day 2, 10 *Ae*. *aegypti* miRNAs showed significant changes in their abundance in response to infection. The maximum fold change (FC) was found in aae-miR-286a, aae-miR-2944b-3p and aae-miR-980-3p with log_2_ FC of -1.82, -1.54 and -1.43, respectively (Table 1). Among all the differentially regulated miRNAs, aae-miR-308-3p showed the most considerable depletion (-3.78) at 7 dpi. These values are comparable with miRNA changes seen after DENV infection [17]. However, our study and the DENV study [17], sequenced miRNAs using RNA extracted from whole mosquitoes. More pronounced changes are likely to be observed when using specific tissues that are infected with virus. Furthermore, comparison of infected and uninfected tissues may be useful in determining tissue-specific versus systemic changes in miRNAs. Only miRNAs aae-miR-2940-3p, which is mosquito specific, and aae-miR-1-5p were significantly enriched in ZIKV-infected libraries at this time point. We spotted less alteration in miRNA profile at 14 dpi libraries despite mosquitoes at this time point having the highest viral load (S2 Fig). Overall, among all the differentially expressed miRNAs due to ZIKV infection, significant declines in miRNA abundances are more pronounced than enrichment. A similar observation was also reported in a previous study with DENV2, where only 4 miRNAs out of 35 modulated miRNAs during the course of infection were enriched in response to DENV infection [17]. Further studies investigating the effect of distinct flaviviruses on miRNA expression in *Aedes* mosquitoes are required to confirm if depletion is a general response to infection. The abundance of a few miRNAs was altered in more than one time point after ZIKV infection including, aae-miR-309a, aae-miR-308, aae-miR-286b, aae-miR-2941 and aae-miR-989.

**Table 1:** Differentially expressed *Ae*. *aegypti* miRNAs upon ZIKV infection.

	Normalized RC (Control)			Normalized RC (ZIKV infected)					
	R1	R2	R3	R1	R2	R3	Log FC	P-Value	FDR
***Day 2***									
miR-263a-5p	36652.15	27181.08	22225.01	19050.03	12791.09	18564.69	-0.772	>0.001	0.010
miR-286a	152.99	113.75	71.87	24.82	11.92	58.29	-1.824	>0.001	0.003
miR-2941	28750.73	29156.66	24196.24	16038.26	14054.41	20628.86	-0.695	0.001	0.011
miR-2944b-3p	728.56	582.92	534.37	233.93	68.96	330.77	-1.542	>0.001	0.009
miR-2944b-5p	9117.94	6211.78	5055.22	3142.95	1189.77	4121.39	-1.270	>0.001	0.009
miR-2946	6408.73	7195.53	5317.72	3947.63	3808.98	4286.01	-0.652	>0.001	0.010
miR-308-5p	533.90	590.62	634.02	1092.08	1236.04	734.92	0.800	0.002	0.034
miR-309a	1617.69	1269.01	1487.04	573.99	242.64	879.29	-1.366	>0.001	0.008
miR-980-3p	54.66	36.44	53.25	16.71	16.41	19.90	-1.431	>0.001	0.003
miR-989	22085.75	47068.61	43795.06	76596.29	88415.35	45462.50	0.898	0.002	0.034
***Day 7***									
miR-286b	361.26	511.03	611.97	326.68	147.02	173.10	-1.198	>0.001	0.008
miR-2940-3p	15150.75	22040.64	17096.52	32250.97	28203.91	35773.85	0.826	>0.001	0.004
miR-2941	40698.57	37427.95	44481.30	31999.67	18418.53	21111.91	-0.777	>0.001	0.014
miR-308-3p	58.77	100.97	350.85	9.03	18.25	9.51	-3.781	>0.001	0.000
miR-308-5p	1338.45	1111.09	1030.21	766.07	772.37	758.71	-0.599	0.001	0.018
miR-375	2793.59	2960.56	3169.38	2186.33	1741.64	1855.77	-0.626	>0.001	0.014
mir-1-5p	7.46	0.29	5.87	17.41	11.71	34.06	2.148	0.002	0.035
***Day 14***									
miR-286b	438.08	547.87	308.77	202.32	276.78	193.60	-0.939	>0.001	0.007
miR-305-5p	8139.34	10259.61	7866.41	6668.66	6079.73	5420.66	-0.531	0.002	0.036
miR-308-3p	196.88	110.89	75.05	28.07	47.80	29.08	-1.862	>0.001	0.000
miR-309a	169.84	200.41	112.85	74.58	65.35	27.69	-1.514	>0.001	0.001
miR-71-5p	1538.88	1539.48	1447.11	1780.10	2389.32	2752.36	0.612	>0.001	0.012
miR-989	76630.42	62358.80	77297.98	108619.53	113002.20	156294.84	0.805	>0.001	0.001

**Fig 2:**
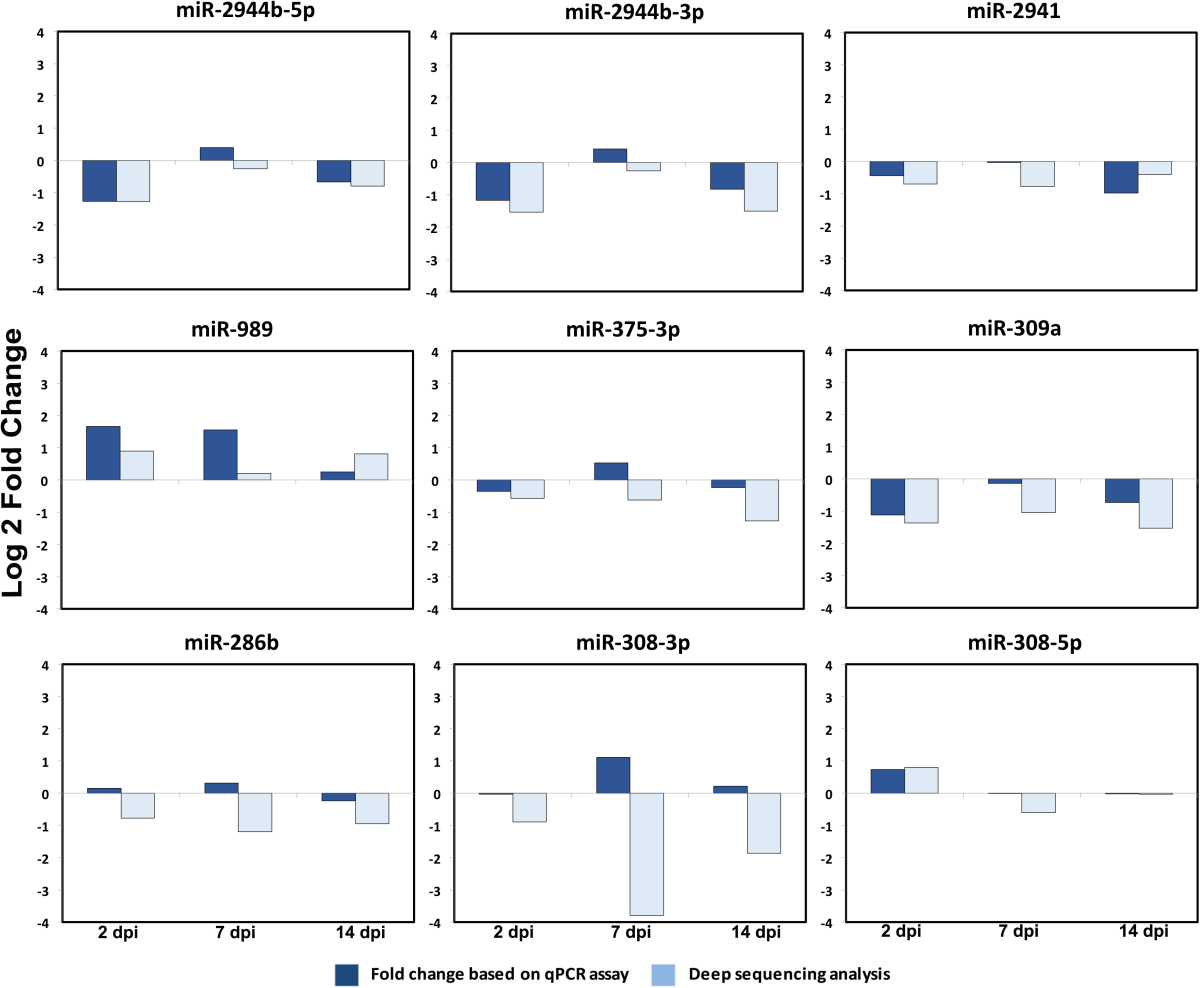
*Ae*. *aegypti* miRNAs are differentially expressed upon ZIKV infection. The graphs show Log_2_ fold changes of a number of *Ae*. *aegypti* miRNAs based on deep sequencing data and RT-qPCR analysis of RNA samples from non-infected and ZIKV-infected mosquitoes at 2, 7 and 14 dpi. Fold changes are averages of three biological replicates.

To validate the differentially expressed miRNAs, nine miRNAs were selected. For this, RNA samples extracted from non-infected and ZIKV-infected whole mosquitoes at 2, 7 and 14 dpi were subjected to miRNA-specific RT-qPCR. Our results showed broad agreement between qPCR and NGS values. While it is not uncommon to find inconsistences between these two quantification approaches [26,27], in 18 out of 27 cases, the direction of gene expression was the same (i.e. both enriched or both depleted) (Fig 2). Where discrepancies were observed, the trend was for NGS data to indicate depletion of the miRNA, while the qPCR suggested no significant changes. A notable inconsistency was seen with the miRNA miR-308-3p that was seen to be enriched by qPCR but depleted by deep sequencing at 7dpi.

A cell line study using *Ae*. *aegypti* Aag2 cells found miRNAs were only mildly affected by DENV infection [28], but in contrast a number of mosquito studies, reported differentially abundant miRNAs in response to a number of arboviruses. However, in most cases, follow up studies to explore the functional significance of those changes and effects on host target genes and virus replication are lacking. Therefore, below we mainly compare the miRNA changes identified in our study with those in previous ones. In *Ae*. *aegypti* mosquitoes infected with DENV2, five, three and 23 miRNAs were differentially expressed at 2, 4 and 9 dpi, respectively [17]. Among those, miR-308-3p and miR-305-5p (9dpi) overlap with those in ZIKV-infected mosquitoes at 7 and 14 dpi; in both host-virus systems both miRNAs showed depletion. In *Ae*. *albopictus* DENV2-infected mosquitoes, overlapping differentially abundant miRNAs with ZIKV-infected mosquitoes from this study are miR-2940-3p (depleted in DENV, but enriched in ZIKV), miR-263a-5p (depleted in both), miR-308-5p (enriched in both), miR-989 (depleted in DENV, but enriched in ZIKV), and miR-2941 (depleted in both) [27]. In another study from the same group with *Ae*. *albopictus* and DENV2 infection specifically in the midgut tissue, three miRNAs (miR-2941, miR-989, miR-2943) were differentially expressed [29], the first two also with change in abundance upon ZIKV infection in this study. Furthermore, miR-989 was found to be depleted in *Culex quinquefasciatus* mosquitoes by 2.8-fold when infected with WNV [30]; although this miRNA was enriched by about 1.8-fold at 2 and 14 dpi with ZIKV in the present study. miR-980 was also differentially expressed in the *Cx*. *quinquefasciatus-*WNV interaction [22].

It appears that the identified differentially expressed miRNAs in different host mosquitoes upon flavivirus infections overlap more with each other than infections with other viruses, such as alphaviruses. For example, none of the major *Ae*. *albopictus* miRNAs that were differentially abundant after CHIKV infection (miR-100, miR-283, miR-305-3p, miR-927) [31] were found among the list of differentially expressed miRNAs from this study; although some of the differentially expressed miRNAs as a result of ZIKV infection could be found among miRNAs showing low levels of differential expression in the CHIKV-mosquito interaction. The similarities in miRNA changes in mosquitoes when infected with flaviviruses as compared to alphavirus infections could be due to (1) antigenic differences between flaviviruses and alphaviruses that may elicit slightly different host responses, or (2) differences in replication strategies; for example, production of subgenomic flavivirus RNA (sfRNA) by flaviviruses, which could function as decoys or sponges against host derived miRNA, suppress the RNAi response, and play other important roles in mosquito-virus interaction [32–34]. Interestingly, sfRNA from WNV has been shown to efficiently suppress siRNA and miRNA-induced RNAi pathways in mosquito cells and its engineering into a Semliki Forest virus (SFV, an alphavirus) replicon led to enhanced replication of SFV in RNAi-competent mosquito cells [32]. While alphaviruses do not produce such RNAs and must rely on other mechanisms to deregulate the host RNAi response.

### Target analysis of differentially abundant miRNAs

The hypothetical binding sites for all the differentially abundant miRNAs upon ZIKV infection were predicted by command line tools miRanda, RNAhybrid and RNA22 v2 using their default parameters. High confidence potential targets were defined as those containing a unique binding site for each miRNA in all the algorithms, with a maximum of 10 nucleotides shifting. We predicted 898 mRNAs, which can potentially be regulated by the differentially abundant miRNAs upon ZIKV infection (S2 Table). Among these predicted target genes, 247 binding sites were identified for aae-miRNA-980-3p while only six predicted binding sites were detected for aae-miR-308-3p. Although this miRNA showed more profound regulation in response to viral infection (day 7), we only identified Rho GTPase as its predicted target gene (S2 Table). Other predicted binding sites for this miRNA are located on coding regions of some hypothetical proteins. Rho proteins are small signaling G proteins, which are involved in a wide range of cellular functions such as cell polarity, vesicular trafficking, the cell cycle and transcriptome dynamics [35]. Among the predicted targets, a number of immune-related genes were found, such as leucine-rich immune protein and Toll-like receptor, possibly indicating the ability of ZIKV to modulate mosquito immunity. While the list of targets provides a catalogue of high confidence targets of *Ae*. *aegypti* differentially abundant miRNAs upon ZIKV infection, further investigations are required to experimentally establish miRNA-target interactions.

Whilst miRNA-target studies have not been carried out on any of the miRNAs reported to be differentially abundant following viral infection in mosquitoes (previous section), except aae-miR-2940-5p, the role of some of these miRNAs are known in other aspects of mosquito or *Drosophila* biology. For example, a number of the differentially expressed miRNAs upon ZIKV infection were also found differentially expressed upon blood feeding in the fat body tissue [36]. These include, miR-308-5p, miR-263a-5p, miR-305-5p, miR-989, miR-2941, miR-286b, miR-2946. miR-309a, specifically was shown to control ovarian development by targeting the Homeobox gene SIX4 [36], and miR-375 was found highly induced in blood fed mosquitoes regulating a number of mosquito genes, including upregulating *cactus* and downregulating *Rel1* [37]. Application of miR-375 mimic in Aag2 cells led to enhanced DENV replication. While this miRNA was found to be mostly depleted after ZIKV infection (Fig 2), it will be interesting to experimentally test if manipulation of this miRNA could have any effect on ZIKV infection by regulating the Toll pathway. In *D*. *melanogaster*, the role of miR-308 in development [38], miR-980 in memory [39], and miR-305 in homeostasis [40] have been reported.

We also screened the ZIKV genome for potential miRNA binding sites of all the 17 modulated miRNAs. Eighty-five possible interactions were identified by three different target predicting algorithms (miRanda, RNAhybrid and RNA22). S3 Table summarizes highly confident binding sites that were predicted by more than one tool. Some miRNAs such as aae-miR263a-5p, aae-miR-286, aae-miR-305-5p, aae-miR308-5p, aae-miR-989 and aae-miR-980-3p can potentially bind to more than one place in the viral genome. Previously, targeting of genomes of RNA viruses by host miRNAs have been reported in mammalian cells [41]. In particular, a number of human miRNAs (hsa-miR-133a, hsa-miR-548g-3p, hsa-miR-223) with potential binding sites in the 5’ and 3’UTRs of different DENV serotypes have been shown to negatively affect replication of the viruses when overexpressed in mammalian cells [42,43]. In mosquitoes, a midgut-specific alb-miR-281 from *Ae*. *albopictus* was shown to target the 5’UTR of DENV2 thereby enhancing replication of the virus [44].

### ZIKV is a target of the *Ae*. *aegypti* RNAi response

Flaviviruses generally produce dsRNA intermediates during their replication, which are the target of their invertebrate host RNAi machinery [10]. The long dsRNAs are recognised and subsequently diced by the ribonuclease Dicer-2 into 21 nt virus-derived short interfering RNAs (viRNAs) that are double stranded and induce the formation of the RNA induced silencing complex (RISC). One of the strands of the duplex is degraded and the other one guides the RISC complex to viral target sequences with complete complementarity. This binding results in the cleavage and degradation of viral RNAs produced during replication of the virus.

**Fig 3:**
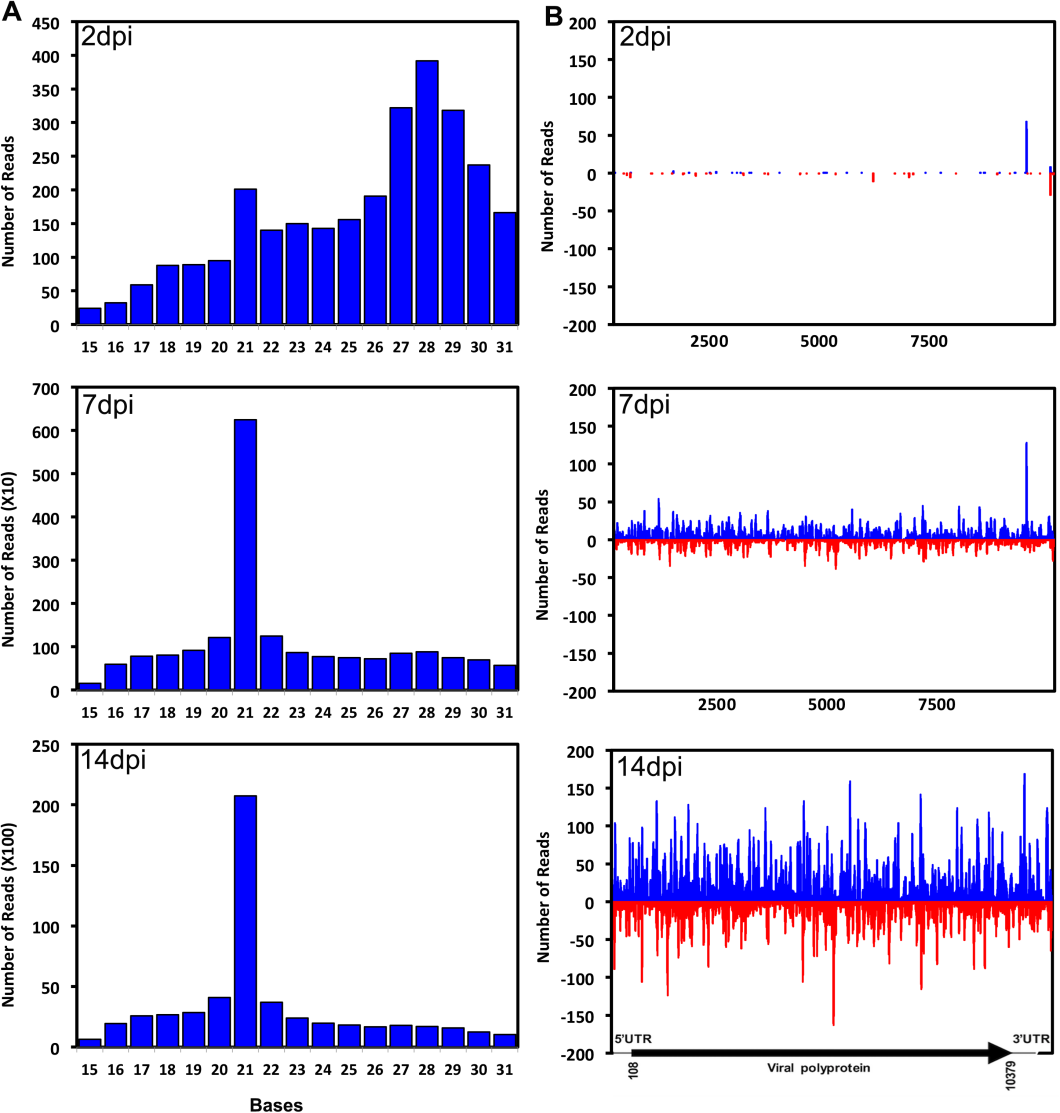
ZIKV elicits an RNAi response in *Ae*. *aegypti* mosquitoes. **(A)** Length distribution of mappable reads to the ZIKV genome in small RNA libraries of *Ae*. *aegypti* mosquitoes at 2, 7 and 14 days post-inoculation (dpi). **(B)** Analysis of virus-derived short interfering RNAs (viRNAs) in *Ae*. *aegypti* ZIKV-infected mosquitoes. Distribution of 21 nt RNA reads that were mapped across the sense (blue) and anti-sense (red) strands of the ZIKV genome at 2, 7 and 14 dpi.

To investigate potential RNAi activity against ZIKV, we mapped all the small RNAs to the viral genome (accession no. KX247632). In total, 3,288, 20,360 and 57,867 reads mapped to the viral genome at 2, 7 and 14 dpi, respectively, ranging in size from 15–35 nt. The total number of reads at 14 dpi that mapped to the virus genome accounted for 0.16% of the total small RNA reads at this time point after infection (36,115,068; S1 Table), which is close to the percentage (0.05%) found in DENV2-infected *Ae*. *aegypti* whole mosquitoes at 9 dpi [45]. The number could possibly be higher if small RNAs are analysed in specific tissues where virus infection primarily occurs. Using whole mosquitoes, which is a mixture of infected and non-infected tissues, may result in dampening of the percentage of virus-specific small RNAs. While at 2 dpi the distribution of small RNAs was across different sizes, at 7 and 14 dpi the majority of the mapped reads were at 21 nt, typical of viRNA size in mosquitoes (Fig 3A). When only the 21 nt reads were mapped to the viral genome, the number of viRNAs increased dramatically during the course of infection; 201 (2 days), 6,250 (7 days), and 20,732 (14 days). This also confirmed successful replication of the virus in the mosquitoes. In addition, the viRNAs mapped across the entire length of the viral genome, on both positive and negative strands of the viral genome (Fig 3B). The pattern of mapped reads indicated a bias towards the positive strand; 62% to the positive strand and 38% to the negative strand–the percentages were very similar both at 7 and 14 dpi. We did not find distinct hot-spots (large number of viRNA production) across the viral genome, except one towards the end of the NS5 region at both 2 dpi and 7 dpi, which is also present at 14 dpi but not as a pronounced peak among others (Fig 3B). These results confirm that ZIKV is exposed to the mosquito host RNAi response, with the replicative dsRNA intermediates being the major substrate for Dicer-2. These findings are consistent with other examples of flaviviruses [28,45–50].

### Production of ZIKV-derived piRNA-like small RNAs

Virus-derived piRNA-like small RNAs (25–30 nt), which are also referred to as viral-derived piRNAs (vpiRNAs), have been identified in insects infected with flavivirues, bunyaviruses and alphaviruses [45,51–54]. It has been shown that knockdown of the piRNA pathway proteins leads to enhanced replication of arboviruses in mosquito cells, suggesting their potential antiviral properties in mosquitoes. For example, knockdown of Piwi-4 in *Ae*. *aegypti* Aag2 cell line increased replication of the mosquito-borne alphavirus, SFV [51]. In another study in the same cell line, specifically silencing Ago3 and Piwi-5 led to significantly reduced production of vpiRNAs against another alphavirus, Sindbis virus (SINV) [55].

**Fig 4:**
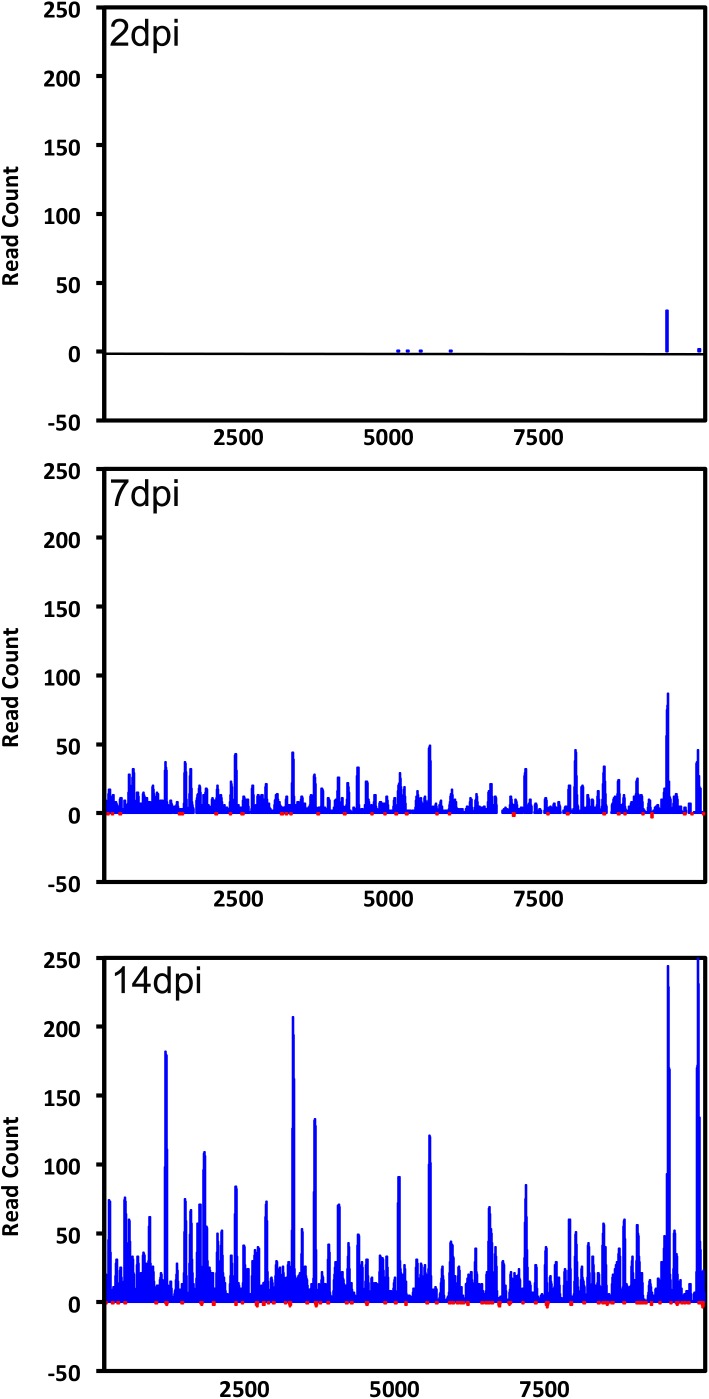
ZIKV-specific piRNA-like small RNAs in infected *Ae*. *aegypti* mosquitoes. Distribution of 24–30 nt small RNAs that mapped across the sense (blue) and anti-sense (red) strands of the ZIKV genome at 2, 7 and 14 dpi.

To find out if any virus-derived piRNA-like small RNAs are produced in *Ae*. *aegypti* mosquitoes infected with ZIKV, we mapped 25–30 nt small RNA reads from the three time points post-infection to the viral genome. The number of reads increased as infection progressed, and they mapped to the entire ZIKV genome with no particular hot spots identified (Fig 4). However, we found a significant bias for reads mapped to the positive strand; for example, in 14 dpi samples 5,300 of 25–30 nt reads mapped to the positive stand and only 60 reads mapped to the negative strand (Fig 4). In DENV2 infected *Ae*. *aegypti* mosquitoes, the number of 25–30 nt reads that mapped to the negative strand of the virus were also extremely low. Further, no bias for a specific base or sequence-specific piRNA signature (U_1_ and A_10_ bias) was observed in this study, as would normally be expected for ping-pong derived piRNAs [56].

Similar observations were reported in other flavivirus-infected mosquitoes or mosquito cell lines. We recently demonstrated that in *Ae*. *aegypti* mosquitoes infected with an insect-specific flavivirus (Palm Creek virus), small RNA reads in the range of 25–30 nt do not harbor any of the classical sequence-specific piRNA features [57]. Hess et al. (2011) also showed that DENV2 piRNA-like sequences do not display any bias for U in the first position and only a slight bias for A_10_ [50]. However, in mosquito cells infected with alphaviruses SFV [51] and SINV [52], and bunyaviruses La Crosse virus [52] and Rift Valley Fever virus [58] clear U_1_ and A_10_ ping-pong piRNA signature was observed. Hence, currently we do not have enough evidence to classify the 25–30 nt reads that mapped to the ZIKV genome as vpiRNA since they might be artefacts of viral genome degradation.

In summary, we found that ZIKV infection in *Ae*. *aegypti* altered the small RNA profile of mosquitoes with peaks seen at 21–22 and 27–29 nt. Overall, ZIKV infection modulated 17 miRNAs with the majority of these small RNAs being depleted. Several immune related transcripts were the predicted targets of differentially abundant miRNAs suggesting that ZIKV may interact with mosquito immunity. At 7 and 14 dpi, viral infection initiated an RNAi response indicated by the presence of viRNAs. At these times points, virus-derived small RNAs in the size range of piRNAs were also found in infected mosquitoes, although they lacked the typical piRNA signature. This study increases our understanding of ZIKV-mosquito interactions and broadens our comprehension of the *Aedes* miRNA response to flavivirus infection.

## Supporting information

S1 Fig Average Ct values of the U6B small nuclear RNA in Zika virus infected and uninfected mosquitoes at days 2, 7, and 14 post infection. (PDF)

S2 Fig Viral quantification of individual mosquitoes used for high throughput sequencing. (PDF)

S1 Table Small RNA read summary in ZIKV-infected and non-infected libraries. (DOCX)

S2 Table Predicted transcripts modulated by miRNAs. (XLSX)

S3 Table Potential interactions between the host differentially expressed miRNAs and the ZIKV genome. (DOCX)

S4 Table Primer sequences for qPCR. (DOCX)
