# Supplementary material for: Zika virus alters the microRNA expression profile and elicits an RNAi response in Aedes aegypti mosquitoes
Source: PLoS Negl Trop Dis. 2017 Jul 17;11(7):e0005760. doi: 10.1371/journal.pntd.0005760 (PMC5531668; doi:10.1371/journal.pntd.0005760)
Supplement: S1 Fig — (PDF) [file pntd.0005760.s001.pdf]

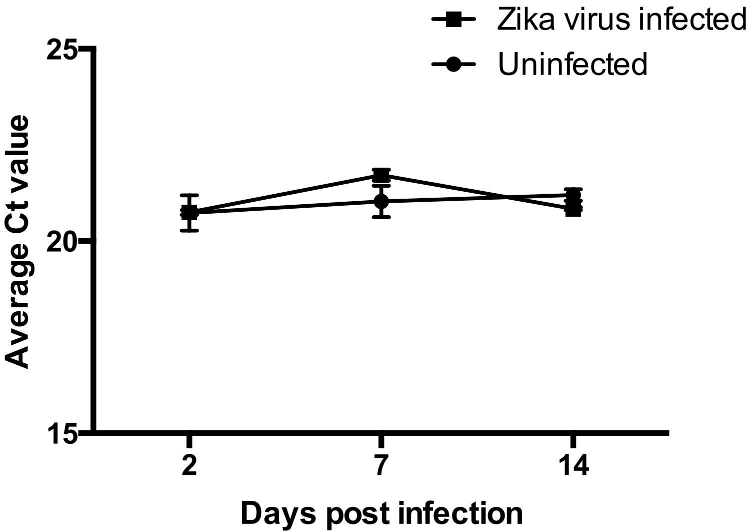

Figure S1. Average Ct values of the U6B small nuclear RNA in Zika virus infected and uninfected mosquitoes at days 2, 7, and 14 post infection. No significant changes were seen in Ct values over time.
