# Supplementary material for: Zika virus alters the microRNA expression profile and elicits an RNAi response in Aedes aegypti mosquitoes
Source: PLoS Negl Trop Dis. 2017 Jul 17;11(7):e0005760. doi: 10.1371/journal.pntd.0005760 (PMC5531668; doi:10.1371/journal.pntd.0005760)
Supplement: S2 Fig — (PDF) [file pntd.0005760.s002.pdf]

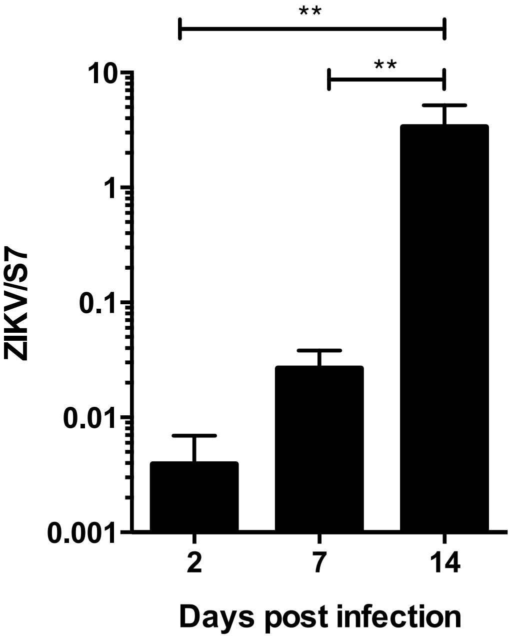

Figure S1. Viral quantification of individual mosquitoes used for high throughput sequencing. Viral titer (ZIKV genomic copies) was normalized to the expression of the host S7 gene. Error bars represent SEM and data were analyzed by Kruskal-Wallis test using Dunn's multiple comparisons test.
