# Supplementary material for: Zika virus alters the microRNA expression profile and elicits an RNAi response in Aedes aegypti mosquitoes
Source: PLoS Negl Trop Dis. 2017 Jul 17;11(7):e0005760. doi: 10.1371/journal.pntd.0005760 (PMC5531668; doi:10.1371/journal.pntd.0005760)
Supplement: S1 Table — (DOCX) [file pntd.0005760.s003.docx]

**Table S1.** Small RNA read summary in ZIKV-infected and non-infected libraries.

| Sample ID | Condition | Number of reads | Number of Clean reads | Percentage trimmed | Total Small RNA (UR)* | Total Small RNA (RC)** | aae-miRNA (UR) | aae-miRNA (RC) |
| --- | --- | --- | --- | --- | --- | --- | --- | --- |
| S1 | Day 2-Mock | 19,324,567 | 16,407,357 | 84.90% | 268,051 | 9,304,264 | 5,106 | 2,511,723 |
| S2 | Day 2-Mock | 19,630,697 | 16,516,747 | 84.14% | 266,870 | 9,304,210 | 5,227 | 2,669,696 |
| S3 | Day 2-Mock | 20,561,100 | 17,385,355 | 84.55% | 273,653 | 9,331,593 | 5,152 | 2,520,999 |
| S4 | Day 2-ZIKV | 19,044,175 | 14,229,706 | 74.72% | 202,798 | 6,783,576 | 3,853 | 1,423,524 |
| S5 | Day 2-ZIKV | 16,069,439 | 12,779,382 | 79.53% | 181,163 | 6,396,453 | 3,723 | 1,420,265 |
| S6 | Day 2-ZIKV | 19,605,636 | 15,681,231 | 79.98% | 223,077 | 7,892,553 | 4,766 | 2,237,466 |
| S7 | Day 7-Mock | 20,516,202 | 17,080,580 | 83.25% | 222,964 | 9,086,313 | 4,140 | 1,948,820 |
| S8 | Day 7-Mock | 19,667,720 | 15,134,458 | 76.95% | 197,667 | 7,512,861 | 3,869 | 1,530,688 |
| S9 | Day 7-Mock | 19,806,270 | 15,692,814 | 79.23% | 215,868 | 8,724,171 | 4,184 | 1,849,535 |
| S10 | Day 7-ZIKV | 22,168,089 | 17,619,389 | 79.48% | 251,544 | 9,339,888 | 5,241 | 2,839,934 |
| S11 | Day 7-ZIKV | 20,953,272 | 17,079,114 | 81.51% | 248,334 | 8,687,874 | 5,023 | 2,513,319 |
| S12 | Day 7-ZIKV | 20,089,224 | 16,915,866 | 84.20% | 248,212 | 9,223,746 | 5,306 | 2,905,769 |
| S13 | Day 14-Mock | 24,152,116 | 20,334,497 | 84.19% | 288,159 | 10,744,949 | 5,138 | 2,663,048 |
| S14 | Day 14-Mock | 19,006,207 | 15,962,816 | 83.99% | 221,321 | 7,581,611 | 4,228 | 1,758,939 |
| S15 | Day 14-Mock | 18,607,685 | 15,922,115 | 85.57% | 235,902 | 8,567,218 | 4,451 | 2,007,202 |
| S16 | Day 14-ZIKV | 23,313,343 | 18,312,742 | 78.55% | 251,365 | 9,310,372 | 4,647 | 2,290,642 |
| S17 | Day 14-ZIKV | 48,248,761 | 38,671,373 | 80.15% | 528,969 | 21,781,331 | 6,740 | 5,112,266 |
| S18 | Day 14-ZIKV | 13,243,550 | 10,055,504 | 75.93% | 119,495 | 5,023,365 | 3,362 | 1,411,184 |

* UR: Unique read sequence

** RC: Read count
