# Supplementary material for: Zika virus alters the microRNA expression profile and elicits an RNAi response in Aedes aegypti mosquitoes
Source: PLoS Negl Trop Dis. 2017 Jul 17;11(7):e0005760. doi: 10.1371/journal.pntd.0005760 (PMC5531668; doi:10.1371/journal.pntd.0005760)
Supplement: S3 Table — (DOCX) [file pntd.0005760.s005.docx]

**Table S3.** Potential interactions between the host differentially expressed miRNAs and the ZIKV genome.

| **miRNA** | **Start** | **End** | **Tool 1** | **MFE**  **(Kcal/mol)** | **Tool 2** | **MFE**  **(Kcal/mol)** | **Binding site** |
| --- | --- | --- | --- | --- | --- | --- | --- |
| miR-263a-5p | 5086 | 5108 | miRanda | -24.21 | RNAhybrid | -26 | 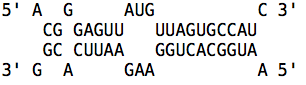 |
| miR-263a-5p | 5382 | 5402 | RNAhybrid | -20.5 | miRanda | -17.71 | 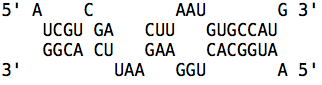 |
| miR-286a | 8105 | 8128 | RNA22v2 | -16 | RNAhybrid | -23.7 | 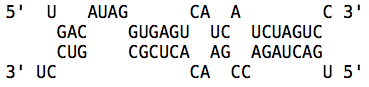 |
| miR-286a | 8105 | 8128 | miRanda | -18.44 | RNA22v2 | -16 | 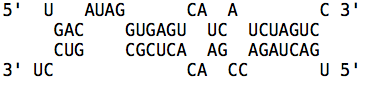 |
| miR-286b | 8108 | 8130 | RNAhybrid | -20.5 | miRanda | -16.72 | 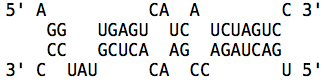 |
| miR-305-5p | 1950 | 1971 | RNA22v2 | -16.8 | RNAhybrid | -23 | 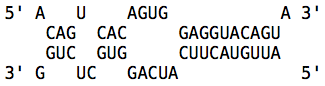 |
| miR-305-5p | 4883 | 4904 | RNAhybrid | -25.3 | RNA22v2 | -18 | 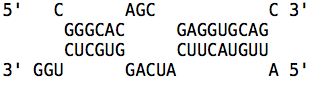 |
| miR-308-5p | 7303 | 7322 | RNAhybrid | -22.7 | RNA22v2 | -21.4 | 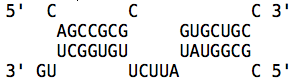 |
| miR-308-5p | 10499 | 10518 | RNAhybrid | -23.4 | RNA22v2 | -17 | 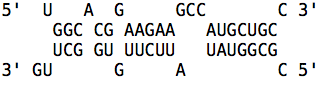 |
| miR-980-3p | 3660 | 3675 | RNA22v2 | -15.6 | RNAhybrid | -21.2 | 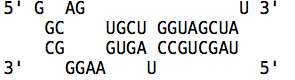 |
| miR-980-3p | 7320 | 7338 | miRanda | -13.19 | RNAhybrid | -23.6 | 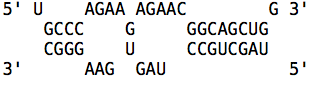 |
| miR-989 | 3979 | 3997 | RNAhybrid | -20.6 | miRanda | -17.59 | 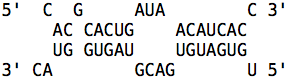 |
| miR-989 | 4388 | 4406 | RNAhybrid | -20.2 | miRanda | -15 | 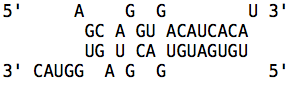 |
| miR-989 | 4753 | 4771 | RNAhybrid | -28.4 | RNA22v2 | -20.7 | 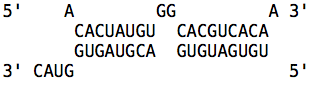 |
