# Supplementary material for: Zika virus alters the microRNA expression profile and elicits an RNAi response in Aedes aegypti mosquitoes
Source: PLoS Negl Trop Dis. 2017 Jul 17;11(7):e0005760. doi: 10.1371/journal.pntd.0005760 (PMC5531668; doi:10.1371/journal.pntd.0005760)
Supplement: S4 Table — (DOCX) [file pntd.0005760.s006.docx]

**Table S4.** Primer sequences for qPCR.

| Primer name | Sequence (5' - 3') | Reference |  |
| --- | --- | --- | --- |
| Zika4481_F | CTGTGGCATGAACCCAATAG | (Waggoner and Pinsky, 2016) | |
| Zika4552c_R | ATCCCATAGAGCACCACTCC | (Waggoner and Pinsky, 2016) |  |
| Zika4507cFAM | -[6FAM]CCACGCTCCAGCTGCAAAGG[BHQ1] | (Waggoner and Pinsky, 2016) |  |
| AaegRps7-F | CGCGCTCGTGAGATCGA | (Sengul and Tu, 2010) |  |
| AaegRps7-R | GCACCGGGACGTAGATCA | (Sengul and Tu, 2010) |  |
| AegRps7 probe | [6FAM]ACAGCAAGAAGGCTATCG[BHQ1] | (Sengul and Tu, 2010) |  |
| aae-miR-2941 | TAGTACGGCTAGAACTCCACGG | This study |  |
| aae-miR-308-5p | CGCGGTATATTCTTGTGGCTTG | This study |  |
| aae-miR-286b | TGACTAGACCGAACACTCGTATCCC | This study |  |
| aae-miR-308-3p | AATCACAGGAGTATACTG | This study |  |
| aae-miR-309a | TCACTGGGCAAAGTTTGTCGC | This study |  |
| aae-miR-989 | TGTGATGTGACGTAGTGGTAC | This study |  |
| aae-miR-2944b-3p | TATCACAGCAGTAGTTACCTGA | This study |  |
| aae-miR-2944b-5p | GAAGGAACTCCCGGTGTGATATA | This study |  |
| aae-miR-375-3p | TTTGTTCGTTTGGCTCGAGTTA | This study |  |
| aae-miR-286a-3p | TGACTAGACCGAACACTCGCGTCCT | This study |  |
| RNU6B | CGCAAGGATGACACGCAAATTCGTGAAGCGTTCCATATTTTT | Qiagen kit |  |
